# Single-port transaxillary robotic thyroidectomy (START): 200-cases with two-step retraction method

**DOI:** 10.1007/s00464-021-08837-9

**Published:** 2021-11-05

**Authors:** Jin Kyong Kim, Sun Hyung Choi, Soon Min Choi, Hye Ryeon Choi, Cho Rok Lee, Sang-Wook Kang, Jong Ju Jeong, Kee-Hyun Nam, Woong Youn Chung

**Affiliations:** 1 Department of Surgery, Yonsei Cancer Center, Severance Hospital, Yonsei University College of Medicine, 50-1, Yonsei-ro, Seodaemun-gu, Seoul, Republic of Korea; 2 Department of Surgery, Yongin Severance Hospital, Gyeonggi-do, Korea

**Keywords:** Robotic thyroidectomy, Robotic transaxillary thyroidectomy, da vinci SP, Robotic single-port thyroidectomy, Robotic single-port transaxillary thyroidectomy, START

## Abstract

**Background:**

This study aims to report the results of a pioneering clinical study using the single-port transaxillary robotic thyroidectomy (START) for 200 patients with thyroid tumor and to introduce our novel two-step retraction method.

**Methods:**

START was performed on consecutive 200 patients using the da Vinci Single-Port (SP) robot system from January 2019 to September 2020 at the Yonsei University Health System, Seoul, Korea. The novel two-step retraction technique, in which a 3.5 cm long incision is made along the natural skin crease, was used for the latter 164 patients. The surgical outcome and invasiveness of the SP two-step retraction method were analyzed.

**Results:**

Among the 200 cases who underwent START, 198 were female and 2 were male, with a mean age of 34.7 (range: 13–58 years). Thyroid lobectomy was performed for 177 patients and total thyroidectomy was performed for 23 patients. Ten patients had benign thyroid nodules, whereas the other 190 had thyroid malignancy. The mean body mass index (BMI) was 22.2 ± 3.7 kg/m^2^ (range: 15.9–37.0 kg/m^2^). All of the operations were performed successfully without any open conversions, and patients were discharged on postoperative day 3 or 4 without significant complication. The mean operative time for thyroid lobectomy with the two-step retraction method was 116.69 ± 23.23 min, which was similar to that in the conventional robotic skin flap method (115.33 ± 17.29 min). We could minimize the extent of the robotic skin flap dissection with the two-step retraction method.

**Conclusions:**

START is a practical surgical method. By employing the new two-step retraction method, we can maximize the cosmetic and functional benefits for patients and reduce the workload fatigue of surgeons by increasing robotic dependency.

**Supplementary Information:**

The online version contains supplementary material available at 10.1007/s00464-021-08837-9.

Minimally invasive robotic surgeries for thyroid cancer and other thyroid diseases are frequently performed using various methods, such as the transaxillary [1], BABA [2], facelift [3], transoral [4], and retroauricular approaches [5]. Our institution strives to develop safe and feasible transaxillary approaches for thyroid surgeries.[1, 6–12].

The recently introduced da Vinci SP® Surgical System (Intuitive Surgical, Sunnyvale, CA, USA) provides access to the narrow and deep portions of internal body organs with minimal incision, which is suitable for indolent diseases such as most thyroid tumours including carcinoma [13, 14]. Young patients have high incidence of thyroid carcinoma with extremely low mortality rates [15, 16]; hence, the quality of life of this patient group after surgery is crucial. In order to reduce postoperative pain, adhesion, and scars, our institution introduced the da Vinci SP system.

The difference between the da Vinci SP system and the previous platforms is that this system has a single trocar that delivers three multijointed instruments and a fully wristed three-dimensional high-definition camera, instead of four distinct robotic arms, which reduces collision between the robotic arms in narrow and confined spaces. In this paper, we introduce our novel surgical technique with a two-step method using the da Vinci SP system, which can increase robotic efficiency and minimize invasiveness.

## Method

### Patients

Single-port transaxillary robotic thyroidectomy (START) was performed on consecutive 200 patients using the da Vinci SP robot system from January 2019 to September 2020. Among them, 36 patients underwent robotic thyroidectomy involving a conventional robotic skin flap method (conventional method) with a 3.5 cm skin incision along the natural skin crease [1]. From the 37th case, we applied the novel two-step retraction technique (two-step method) to minimize flap invasiveness and to obtain a stable view.

The preoperative evaluation of all the thyroid nodules included fine needle aspiration biopsy (FNAB). When thyroid carcinoma was suspected, high-resolution staging ultrasonography and neck and chest computed tomography (CT) were performed. The ultrasound examinations included both thyroid lobes and all the neck levels (I–VI) [17]. For benign tumours, surgical management was indicated as follows: (1) follicular/Hurthle cell lesions of diameter < 5 cm and (2) palpable benign tumors of diameter ≥ 4 cm.

Cases of thyroid nodules without lateral neck or distant metastasis were enrolled in this study; those involving tumours with posterior capsular invasion or extension to an adjacent structure were excluded due to the possibility of injury to the recurrent laryngeal nerve (RLN), trachea, or oesophagus during the procedure. Cases with goitre or Grave’s disease were also excluded due to the high risk involved.

In thyroid cancer, the extents of thyroidectomy were determined according to the guidelines issued by the 2015 American Thyroid Association [18]. Twenty-three patients with bilateral lesions, adjacent muscle invasion during surgery, or more than five central lymph node metastasis underwent total thyroidectomy. Prophylactic ipsilateral central compartment neck dissection was performed for all the cancer patients.

Details on the clinical and pathologic characteristics, operation type, operative time, postoperative hospital stay, and complications were obtained from our institutional database. This study was approved by Yonsei University’s institutional review board (4–2020-0149), and informed consent from the patients was waived owing to the retrospective nature of this study.

### Operative procedure

#### Skin flap creation with the conventional method

Under general anaesthesia, the patient was placed in a supine position. A backrest was placed underneath the patient to allow neck extension in order to provide a fully exposed surgical space to the surgeon. The arm on the lesion-side was not fixed but draped to allow movement so that it could be raised or stretched laterally during the operation. Generally, the lesion-side arm was raised during flap dissection and regulated as occasion demands during the robotic console time. A 3.5 cm incision was made along the natural skin crease. Flap dissection was performed as in general robotic transaxillary thyroidectomy. The detailed procedure has been described in previous studies [6, 19].

#### Skin flap creation with the two-step method

**Fig. 1:**
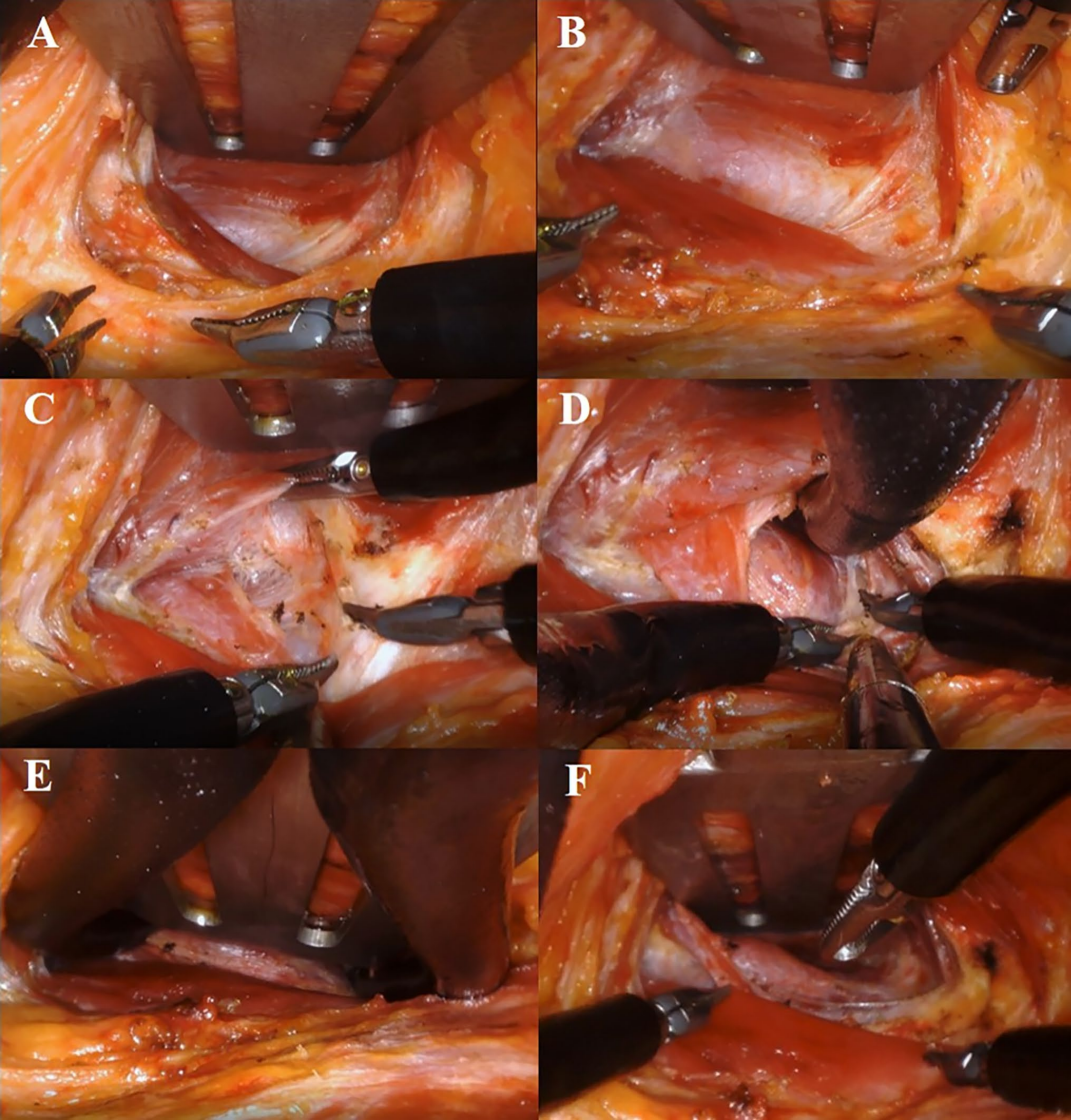
Two-step retraction operative procedure. **A** Robotic console view after the first docking. **B** Widening of the skin flap with the da Vinci SP. **C** Working space creation between the two heads of the SCM. **D** Finding thyroid beneath the strap muscles. **E** The second docking creating sufficient working space. F: Completion of the second docking

The preoperative patient position was the same as that in the conventional method. A 3.5 cm incision was made along the natural skin crease on the lesion-side axilla. With the arm raised, flap dissection was performed over the anterior surface of the pectoralis major muscle, directly toward the sternal notch to find the sternocleidomastoid muscle (SCM). After the SCM was identified below the platysma and its two heads dissected, a narrow (width = 3 cm) version of the Chung’s retractor was inserted, corresponding to the size of the incision. The skin with a small portion of the SCM sternal head was then elevated. The raised arm was stretched laterally to straighten the robotic approach around the posterior neck area. After robotic docking, the subplatysmal layer was further dissected to obtain sufficient space. The two heads of the SCM were then dissected completely to find the strap muscles. The thyroid was found below the strap muscles and after obtaining sufficient space, the surgical assistant inserted the Chung’s retractor below the strap muscles, completing the redocking; this is referred to as “the second step retraction.” The step-by-step robotic procedure after the first docking is shown in Fig. 1 and video.

### Robotic thyroidectomy technique

Thyroidectomy was performed as in conventional transaxillary robotic surgery [6]. The robotic camera was employed at the bottom of the SP trocar. The three-arm method was used: Cardiere on the upper site, and two Maryland dissectors on the left and right of the single-port trocar. As the bipolar energy source, Erbe (Erbe USA Inc, Marietta, GA) in the SWIFT COAG mode at a power level of 4–5 was connected to the bilateral Maryland dissectors. Fine upper-pole dissection was performed saving the external branch of RLN and superior parathyroid. Further, the ipsilateral RLN was identified and central compartment dissection was simultaneously performed on the cancer patients. While tracing the nerve, the ipsilateral thyroid was detached from the trachea and a specimen was extracted through an axillary incision. The detailed thyroid lobectomy procedure has been presented in a previous study [20].

For total thyroidectomy, intraoperative nerve monitoring was routinely applied to reduce the risks of RLN injury [21]. Contralateral thyroidectomy was performed after the extraction of the ipsilateral specimen to acquire more working space for the robotic arms. The trachea was pushed down with a suction assistant, the contralateral upper-pole was drawn downward using the robotic grasper, and the superior thyroidal vessel was identified and ligated. After upper-pole dissection, all of the strap muscles around the remnant thyroid were dissected. The contralateral RLN was then identified between the remnant thyroid and the trachea was pushed down by the suction assistant. The RLN was traced above the trachea and detached as a specimen. After the extraction of the contralateral thyroid through an axillary incision, a closed suction drain was inserted for prevention of emergent bleeding or seroma. The wound was closed layer-by-layer.

### The role of an assistant

During the whole procedure, one assistant (a field surgeon or a physician assistant(PA)) joined the operation. When creating the robotic flap, the assistant helped retract the skin flap with specially designed fiber optic device. The assistant also supported the installation of Chung’s retractor, robotic docking, and regulation of robotic location after docking. In the case of the two-step method, the assistant helped loosen the Chung’s retractor and indwell the retractor again beneath the strap muscle in the operation field. Also, with a 50 cm curved suction catheter, the assistant aided in counter-traction, smoke emission, and securing the trachea during the whole console time.

### Postoperative management

All patients were fitted with a drain to prevent complications from postoperative seroma or hematoma and were discharged on the third or fourth postoperative day(POD) after drain removal. After surgery, all of the patients received levothyroxine to maintain thyroid function or to suppress the TSH level, as required. Hypoparathyroidism, defined as reduction in the serum iPTH level below the normal range regardless of hypercalcaemic symptoms [22], was managed with calcium replacement on occurrence.

### Statistical analysis

Details on the clinical characteristics, pathological characteristics, operation data, and complications were obtained from our institutional database. The parameter differences were analysed using student t-tests when appropriate. All statistical analyses were performed using the IBM SPSS Statistics for Windows software (Version 25.0; IBM Corp., Armonk, NY), and the statistically significant difference was defined as *p* < 0.05.

## Result

All operations, including thyroid lobectomy or total thyroidectomy, were performed by a single surgeon (Kee-Hyun Nam). Table 1 lists the clinical characteristics of the 200 patients who underwent START and the pathologic results of 190 patients with thyroid cancer. Among the 200 cases, 198 were female and 2 were male, with a mean age of 34.7 (range: 13–58 years). Thyroid lobectomy was performed for 177patients and total thyroidectomy was performed for 23 patients. Ten patients had benign thyroid nodules, whereas the other 190 had thyroid malignancy. The mean body mass index (BMI) was 22.2 ± 3.7 kg/m^2^ (range: 15.9–37.0 kg/m^2^). Except for two patients who wanted to prolong the hospital stay for four days after the operation, patients were discharged on the third POD.

**Table 1:** Clinical characteristics of patients (*n* = 200) and pathologic result of patients with thyroid cancer (*n* = 190)

Variables	Mean ± SD	Range
Age (years)	34.7 ± 7.9	13–58
Sex ratio (male:female)	2:198	
Extent of thyroidectomy (lobectomy: BTT)	177:23	
Pathologic type (malignant:benign)	190:10	
BMI	22.2 ± 3.7	15.9–37.0
Postoperative hospital stay (days)	3.0 ± 0.1	2–4
Tumor size (cm)	0.9 ± 0.8	0.2–5
Pathology, *n* (%)		
Papillary carcinoma	187 (98.4)	
Follicular carcinoma	3 (1.6)	
Multifocality, n (%)	32 (16.6)	
Bilaterality, n (%)	20 (10.4)	
Central lymph node, n		
Retrieved	3.6 ± 3.3	0–18
Metastatic	0.8 ± 1.3	0–6
T stage, n (%) T1/T2/T3/T4	171/13/5/1	
N stage N0/N1a	117/73	
TNM stage, n (%) I/II/III/IV	190/0/0/0	

The pathologic characteristics of the 190 patients with thyroid cancer were analysed. The mean tumour size was 0.9 ± 0.8 (range: 0.2–5) cm; 187 patients had papillary thyroid carcinoma (PTC) and 3 had minimally invasive follicular thyroid carcinoma. Thirty-two patients had multifocality (> 1 PTC foci on one side of the thyroidectomy specimen), and twenty had bilaterality. The number of retrieved central lymph nodes (CLNs) was 3.6 ± 3.3 (range: 0–18) and the number of metastatic CLNs was 0.8 ± 1.3 (range: 0–6).

The operative data of thyroid lobectomy cases with START were compared between the conventional method and two-step method (Table 2). Mean total operative time for thyroid lobectomy using conventional method group and two-step method group were 115.3 ± 17.3 and 116.7 ± 23.2 min, respectively (*p* = 0.700). The working space, docking, and console time were 34.1 ± 7.5 and 28.9 ± 10.9 min (*p* = 0.007) 5.2 ± 1.7 and 3.8 ± 2.0 min (*p* = 0.000), and 48.0 ± 14.2 and 60.0 ± 18.5 min (*p* = 0.000), respectively. Operation time for total thyroidectomy was also calculated. All of the 23 total thyroidectomy cases were performed with two-step method. The total operation time, working space, docking, and console time were 176.6 ± 21.1, 30.3 ± 11.0, 4.9 ± 1.8, and 115.4 ± 19.9 min, respectively.

**Table 2:** Differences in thyroid lobectomy approaches

	Lobectomy with conventional skin flap (*n* = 36)	Lobectomy with double-docking method (*n* = 141)	*p*-value
Total OP time (min)	115.3 ± 17.3	116.7 ± 23.2	0.700
Working space (min)	34.1 ± 7.5	28.9 ± 10.9	0.007
Docking (min)	5.2 ± 1.7	3.8 ± 2.0	0.000
Console (min)	48.0 ± 14.2	60.0 ± 18.5	0.000

*N* number, *OP* operation

Two patients had transient hypoparathyroidism that recovered completely after 2 months of calcium replacement. There were no cases of permanent hypoparathyroidism, RLN injury, seroma, or bleeding.

**Fig. 2:**
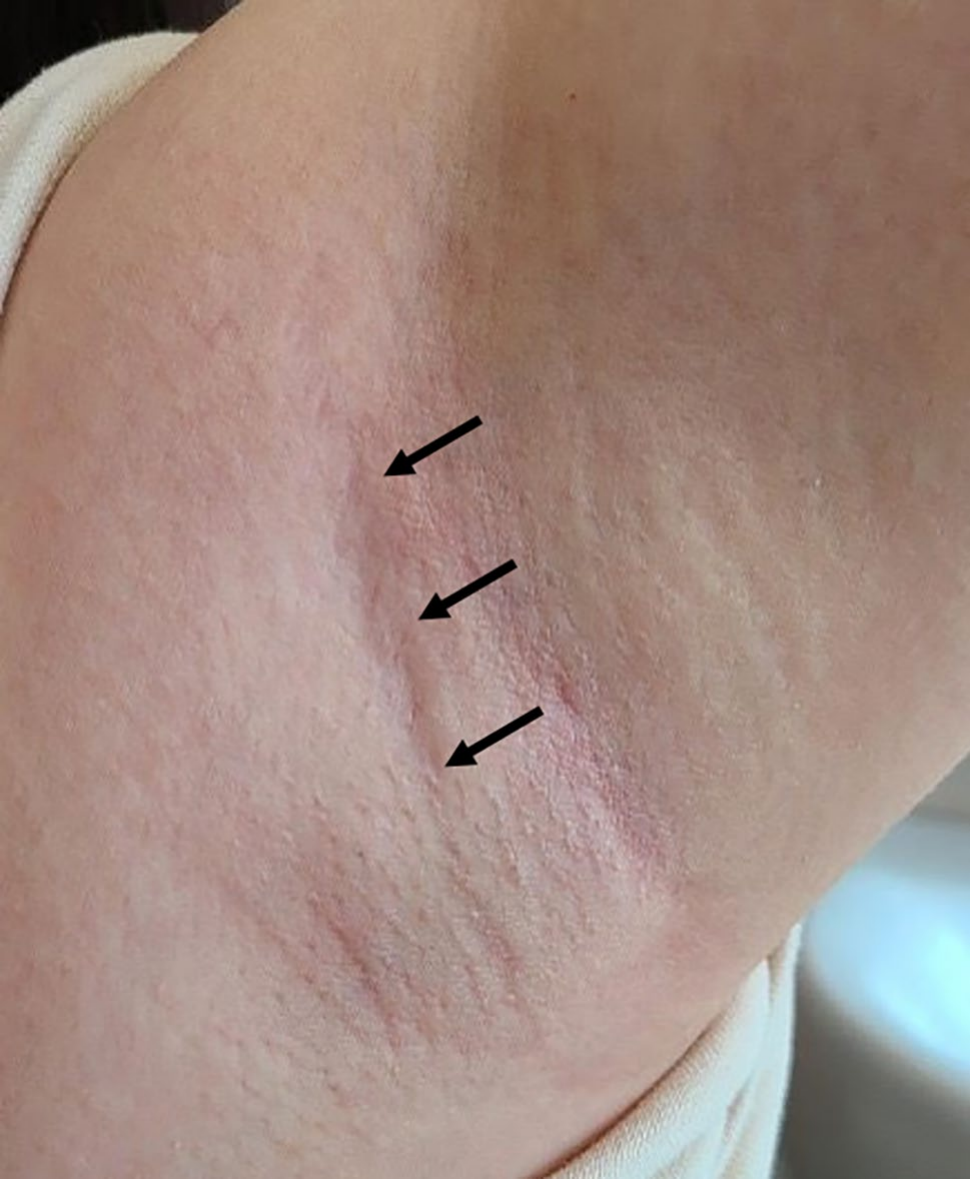
Cosmetic outcome 6 months after surgery

One of the postoperative scars after 6 month from the surgery is provided in Fig. 2. After the complete healing, it became almost indistinguishable from the previous natural skin crease before the operation.

## Discussion

The da Vinci SP platform was originally designed for procedures involving deep and narrow working spaces. Since the development of the robot-assisted endoscopic thyroid surgery by Chung et al. [23] in 2007, our institution has actively performed more than 7500 procedures (July 2020) using the da Vinci Si or Xi platforms. With the introduction of the SP system, however, our institution has considered the possibility of reducing invasiveness through this method.

The da Vinci SP system has been used for thyroid surgery at the Yonsei University Severance Hospital, Seoul, Korea since December 2018. For the first four cases in 2018, the gas insufflation method was employed [20]. However, several layers of cervical muscles hindered the approach to the thyroid from the axilla, demanding considerable effort and time with an unstable view. Hence, we reverted to the gasless method and performed additional 200 START. Thus far, to the best of our knowledge, this is the first study to perform as many as 200 thyroidectomy cases using the da Vinci SP platform.

**Fig. 3:**
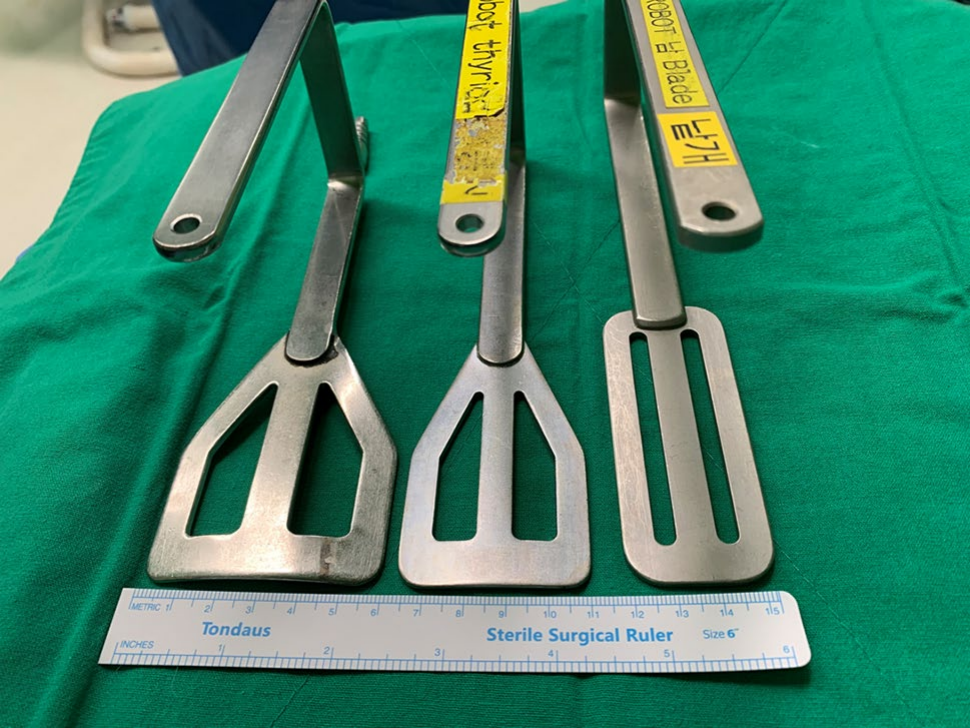
Various version of Chung’s retractors. Currently, the minimal sized Chung’s retractor (right) is used for two-step method

Reduction of the length of the incision was initially attempted using this new apparatus [20]. A narrow version of the Chung’s retractor (Fig. 3) was developed and fibre optics were used during flap dissection. Moreover, the skin flap for robotic thyroidectomy became inevitably narrow due to visual restriction. We realized that upper-pole dissection with a narrow flap was simpler with a laterally extended arm than an upwardly raised one. With the previous Si or Xi platform, we positioned the arm to be upwardly raised in order to ensure least distance from the axilla to thyroid, prohibiting collision between the four robotic arms [1]. Using the SP system, the distance between the axilla and thyroid was no longer a problem.

Nonetheless, longer distance with a small incision was troublesome when creating the working space. The retractor applied during flap dissection could not reach the surface of the thyroid beneath the strap muscles. Hence, we did not fix the lesion-side arm but left it free to allow movement during surgery. We raised the arm during flap dissection and extended it laterally during the console time. When intense concentration was needed on the central compartment, we again raised the arm to facilitate the direct linear approach.

The other problem was that dissection of the skin flap for robotic thyroidectomy around the strap muscles was not visually safe due to the minimization of the incision. We could not easily secure a clear view around the strap muscle with a 3.5 cm incision. Although there were no specific complications, we recognized the necessity for improving the flap dissection. Hence, we employed the two-step method, where we dissected only half the flap, applied docking, completed flap dissection with the robotic console, and redocked the SP robot.

In addition to facilitating a deep and narrow approach, the other advantage of the SP is the ease of docking, de-docking, and redocking. In the Xi or Si systems, four robotic instruments and four robotic trocars had to be moved by adjusting the joints of the four robotic arms, while in the SP, with all the instruments within the trocar, only the single-port trocar needed to be moved. This made the two-step procedure possible without any delay in the operative time.

For the surgeon, the effect of two-step method was apparent; the fatigue of the surgeon and the assistant during flap dissection was reduced. Instead of focusing on retracting all the strap muscles upward, we were able to work between the muscles by spreading the strap muscles with the robotic console, which allowed sufficient working space for the operation. This is also significant since robotic dependency has increased during robotic thyroidectomy.

**Fig. 4:**
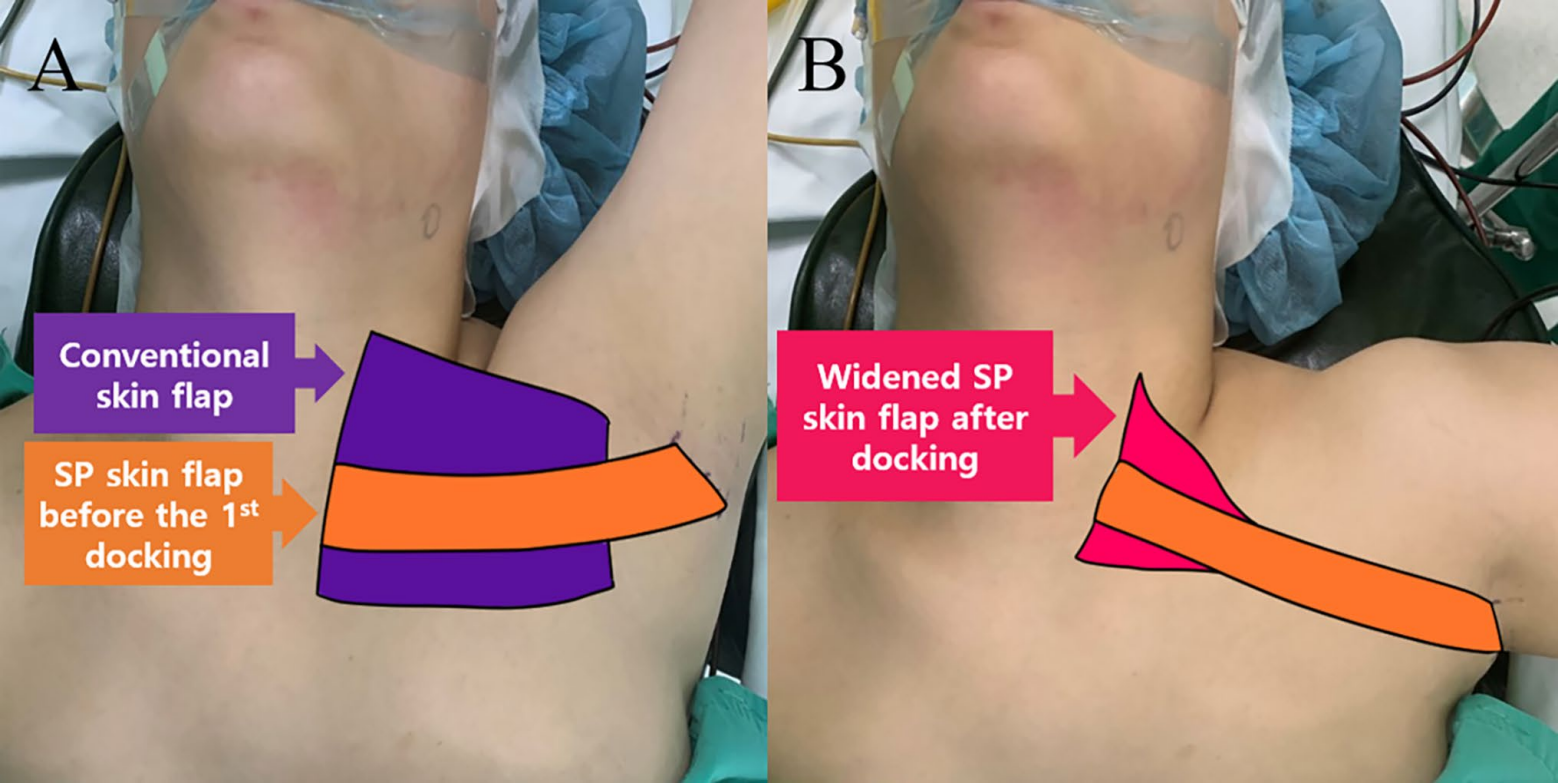
Comparison of the extent of skin flap dissection and the cosmetic outcome. **A** Comparison between conventional skin flap and SP skin flap before docking. **B** Widened SP skin flap after the 1st docking in the two-step method

Moreover, not only the incision length but also the extent of flap dissection for thyroidectomy was decreased using the SP system (Fig. 4A, B). With considerable time and effort, we realized that an entire conventional skin flap for robotic thyroidectomy was not necessary for thyroidectomy with the SP system.

**Fig. 5:**
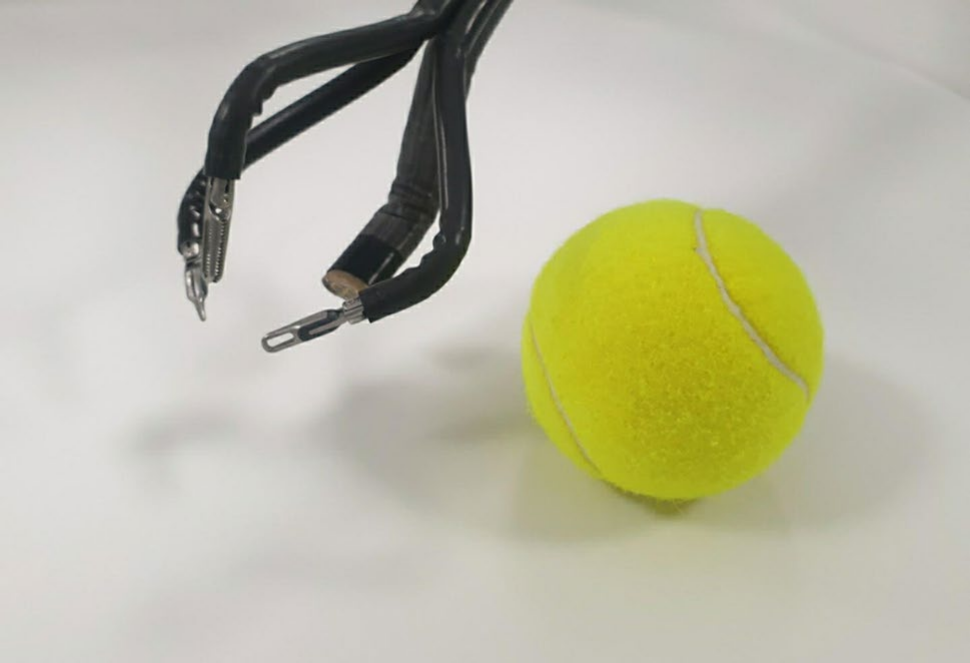
Spherical working space required for the SP system

Although we were comfortable using four linear robotic instruments initially, with increasing experience over time, we became accustomed to the haptic, flexible SP instruments and could freely manipulate the SP system and continuously attempt to render a narrower flap. With the current extent of the robotic transaxillary flap using the SP system, we are uncertain about the benefits of other robotic approaches such as BABA approach, which requires four incisions including two axillary incisions,(2) or transoral thyroidectomy,(4) which occasionally needs an axillary incision for thyroid removal and drain insertion. Furthermore, for the SP system, a spherical space needs to be controlled without restriction (Fig. 5). Beneath the skin, we believe that the transaxillary flap is advantageous for creating sufficient working space for the SP system, without encountering the bone structure.

By applying changes in the patient position during the operation, injury to the brachial plexus or radial, ulnar, and median nerve injuries was avoided in western patient populations [24–29]. If raising the lesion-side arm is not possible, we recommend a laterally stretched arm with the use of longer fibre optics during flap dissection to reach the SCM from the axillary incision.

Currently, a flexible ultrasonic vibration energy device such as the harmonic scalpel is not available due to technical difficulties, which ensued the da Vinci SP system to be equipped with the Erbe. Despite the unfamiliarity with the new energy source, no specific complication occurred in our 200-case experience, and it is noteworthy that there are benefits from the usage of the Erbe compared to the harmonic scalpel. In the SP system, both sides of the flexible Maryland dissectors can be connected to the Erbe and every space of the surgical field can be easily accessed without considerable effort. Moreover, the sharp Maryland forceps facilitate sophisticated dissection, while tracing the nerve and detaching the berry ligament from the nerve without significant thermal injury. Although we could not provide the statistical data due to the retrospective nature of this study, the quality of the patient’s voice after the START is significantly outstanding compared with that treated with any other energy device. The comparison of thermal damage between bipolar energy and ultrasonic energy devices are controversial.[30–41]. Among them, Sutton et al.[42] reported that bipolar energy produce smaller increase in the temperature than harmonic scalpel. Due to the sharp applicators (Maryland) and multiple, short firing of the bipolar energy, we believe we could minimize the thermal damage. This particular subject will be further investigated hereafter.

Our study has revealed satisfactory results with a large sample of 200 patients who underwent START using the da Vinci SP system. As expected, the axillary scar became almost indistinguishable over time (Fig. 2). We assume that this is due to the fact that we used the natural skin crease [43–46]. However, the retrospective nature of the analysis, single surgeon experience, and lack of comparative data for open or other surgical platforms are the limitations of the study. Furthermore, long-term oncological outcomes such as the survival, recurrence, and quality of life need to be evaluated. Therefore, multi-centre prospective studies are required to further evaluate the role of the da Vinci SP platform in thyroid surgeries.

We can conclude that START with the da Vinci SP system is a practical surgical method with minimal invasiveness, particularly when the two-step method is applied. This method offers cosmetic and functional benefits to the patients and reduces the workload fatigue of surgeons.

## Supplementary Information

Below is the link to the electronic supplementary material.

Supplementary file1 (MP4 492743 KB)
